# Collagen Structure-Function Mapping Informs Applications for Regenerative Medicine

**DOI:** 10.3390/bioengineering8010003

**Published:** 2020-12-29

**Authors:** James D. San Antonio, Olena Jacenko, Andrzej Fertala, Joseph P.R.O. Orgel

**Affiliations:** 1 Biocorda LLC, Media, PA 19063, USA; 2 Department of Biomedical Sciences, School of Veterinary Medicine, University of Pennsylvania, 3800 Spruce Street, Philadelphia, PA 19104, USA; jacenko@vet.upenn.edu; 3 Department of Orthopaedic Surgery, Sidney Kimmel Medical College, Thomas Jefferson University, 1015 Walnut Street, Philadelphia, PA 19107, USA; andrzej.fertala@jefferson.edu; 4 Department of Biology, Illinois Institute of Technology, Chicago, IL 60616, USA; orgel@iit.edu; 5 Department of Biomedical Engineering, Illinois Institute of Technology, Chicago, IL 60616, USA; 6 Pritzker Institute of Biomedical Science and Engineering, Illinois Institute of Technology, Chicago, IL 60616, USA

**Keywords:** type I collagen, type III collagen, interactome, microfibril, ligand binding, extracellular matrix, connective tissue, fibrosis, angiogenesis, hemostasis, therapeutic antibodies

## Abstract

Type I collagen, the predominant protein of vertebrates, assembles into fibrils that orchestrate the form and function of bone, tendon, skin, and other tissues. Collagen plays roles in hemostasis, wound healing, angiogenesis, and biomineralization, and its dysfunction contributes to fibrosis, atherosclerosis, cancer metastasis, and brittle bone disease. To elucidate the type I collagen structure-function relationship, we constructed a type I collagen fibril interactome, including its functional sites and disease-associated mutations. When projected onto an X-ray diffraction model of the native collagen microfibril, data revealed a matrix interaction domain that assumes structural roles including collagen assembly, crosslinking, proteoglycan (PG) binding, and mineralization, and the cell interaction domain supporting dynamic aspects of collagen biology such as hemostasis, tissue remodeling, and cell adhesion. Our type III collagen interactome corroborates this model. We propose that in quiescent tissues, the fibril projects a structural face; however, tissue injury releases blood into the collagenous stroma, triggering exposure of the fibrils’ cell and ligand binding sites crucial for tissue remodeling and regeneration. Applications of our research include discovery of anti-fibrotic antibodies and elucidating their interactions with collagen, and using insights from our angiogenesis studies and collagen structure-function model to inform the design of super-angiogenic collagens and collagen mimetics.

## 1. Introduction

Collagens are among the most ubiquitous and complex of the vertebrate extracellular matrix (ECM) macromolecules [1,2,3,4,5]. About thirty genetically distinct collagens are expressed in human connective tissues. For most, the majority of their sequences exist as triple helices, which makes them unique among proteins. Triple helices are rigid, rope-like protein conformations which, depending on the collagen type, may be interspersed between small flexible non-triple helical regions, or larger, globular non-collagenous regions. The triple helical regions are composed of contiguous Gly-X-Y tripeptide repeats, with Gly residues being supported at this position because they are small enough to fit the confines of the three peptide chains that form the triple helix. The extent of the triple helical region, along with the presence of non-triple helical regions, determines the type of aggregate collagen molecules make, and how they contribute to the intricate ECM scaffold that makes up the internal architecture of the vertebrate body.

Type I collagen is the prototypical collagen that aggregates into fibrils. It is the most abundant protein in the human body, comprising about 7 kg of the dry weight of the human adult. There are approximately 1 × 10^23^ collagen molecules in the human body [6]. Remarkably, if the collagen molecules from an adult human were laid end to end, the resulting rope would be long enough to lasso the Moon from the Earth many times over, or easily span the distance between the Earth and the Sun [7]. That so many collagen molecules are packed tightly within one organism is a testament to the exquisite and efficient way vertebrate cells assemble and twist collagen molecules into rope-like fibrils—the most abundant molecular aggregate formed by collagens in vivo.

Type I collagen comprises much of the substance of connective tissues including tendon, ligaments and skin, and most of the organic phase of bone, and supports and provides form to many other tissues of the vertebrate body via the connective tissue proper [1,3,8]. In bone, the type I collagen fibril also serves as the site for mineralization either directly, or by its association with mineralization nucleation proteins [9,10,11,12]. It is therefore no surprise that type I collagen plays crucial roles in vital physiologic processes, including hemostasis, angiogenesis, and biomineralization, and in human pathologies including cancer, fibrosis, and atherosclerosis [3,13,14]. Type I collagen from animal sources is also the most widely used biomaterial for fabrication of bone regeneration scaffolds, hemostats, bandages, and tendon repair patches [15]. Therefore, from both the basic and applied standpoints, it is of paramount interest to understand collagen biology and define the collagen structure-function relationship. Here we will review our research on creating and analyzing type I collagen and type III collagen interactomes, and our use of computational approaches to model these collagens’ molecular interactions with cells, bioactive factors, and other macromolecules. Further, we will discuss how our findings inform applications including the design and discovery of anti-fibrotic and pro-angiogenic therapies.

## 2. Type I Collagen Structure and Assembly

**Figure 1:**
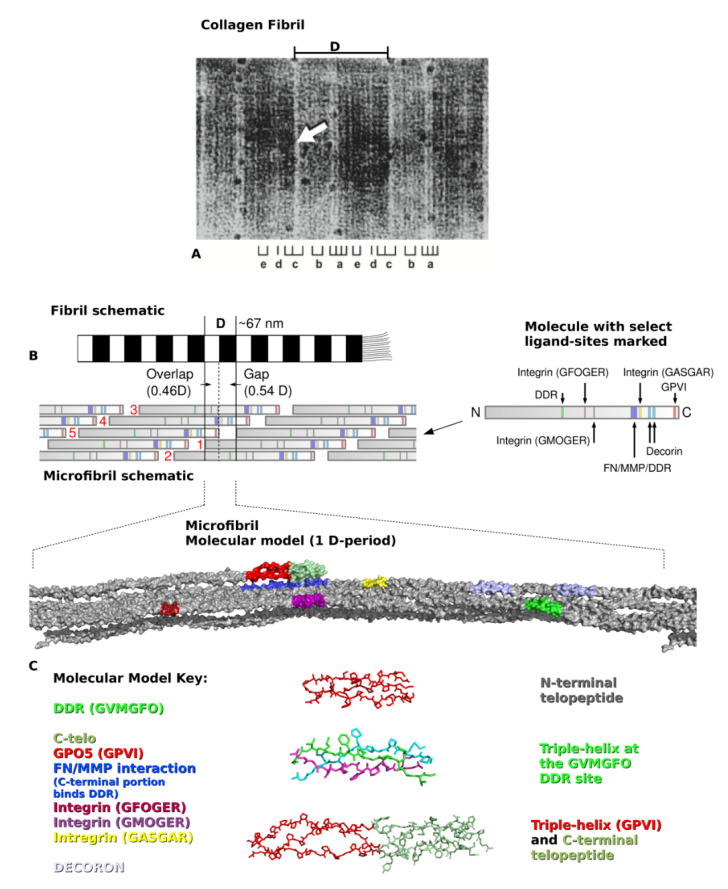
Type I collagen assembly and structure. (**A**). Segment of Type I collagen fibril visualized by transmission electron microscopy. One molecular repeat or D-period (D) is indicated. The positive stain microscopy bands, a–e, are as indicated below the image; arrow indicates left border of overlap zone. This fibril preparation was used to localize heparin-binding sites; thus, heparin-gold particles appear as dark circles bound to the fibril. Originally published in San Antonio et al., 1994, *J. Cell Biol*., *125*, 1179–1188. (**B**). Fibril schematic depicted as negatively-stained TEM preparation where gap regions are dark and overlap regions light. Microfibril schematic shows the Hodge-Petruska scheme [20] of packing where collagen molecules (numbered 1–5 for molecular (M) segments as in M1, M2, etc.) are staggered so that every five M segment does not traverse the entire D-period. Select collagen functional domains (right) are marked along the length of the collagen molecule. (**C**). A single D period of a single microfibril is shown beneath the microfibril schematic. The C-terminal telopeptide (marked in green on the top of the microfibril) and the rest of monomer 5 is orientated towards the outside of the fibril. The side view is from an observers’ perspective from a neighboring microfibril. Note the molecular segments are relatively straight in the overlap zone but re-organize towards the end of the gap zone especially in the region of the supertwist in the vicinity of the gap zone’s discoidin domain receptor 2 (DDR2) binding site. Figure segments reprinted with permission from [21].

Type I collagen is synthesized by cells as pro-α1 and pro-α2 procollagen chains, encoded by separate genes and comprising about 1000 amino acid residues each [2]. The C-terminal propeptides promote the polymerization of two pro-α1 and one pro-α2 chains into the triple helical procollagen molecule. Extracellularly, the globular termini of procollagen are removed by proteolysis, yielding trimeric collagen monomers of a little over 300-nm long. Five monomers assemble in a quarter-staggered fashion to form the microfibril, the basic subunit of the collagen fibril (Figure 1). Specifically, along the fibril’s long axis exists a repeating D-period pattern of molecular organization. Within the 67-nm D-period, groups of five neighboring collagen molecules wind around each other into microfibrils that interdigitate with neighboring microfibrils to form practically inseparable connections within the fibril. The amino acid sequences of the single collagen molecules are found within each five-molecule segment that defines each D-period. Because ~300 nm (collagen triple helix length)/67 nm (D-period length) produces a non-integer number, the D-period contains a region of incomplete overlap, called the gap zone. This space within the microfibril plays a role in the biomechanical properties of the fibril. The gap region also allows biomineralization of bone by accommodating hydroxyapatite crystal formation and growth [16,17]. Because of the gap zone, the remaining four full-length segments arrange around each other forming a twist that somewhat mirrors the superhelix of the collagen molecule, albeit on a larger scale [18]. Each microfibril (Figure 1B,C), and its neighbors may be connected by N- and C-terminal intermolecular cross-links, yet, collagen fibrils exhibit varying degrees of crosslinking depending on tissue location, age, and other circumstances [3]. Other collagen types, proteoglycans (PGs), and matrix macromolecules typically assemble onto the fibril to impart tissue-specific properties to the polymer [3,19].

## 3. Creating a “Road Map” or Interactome of Type I Collagen

**Figure 2:**
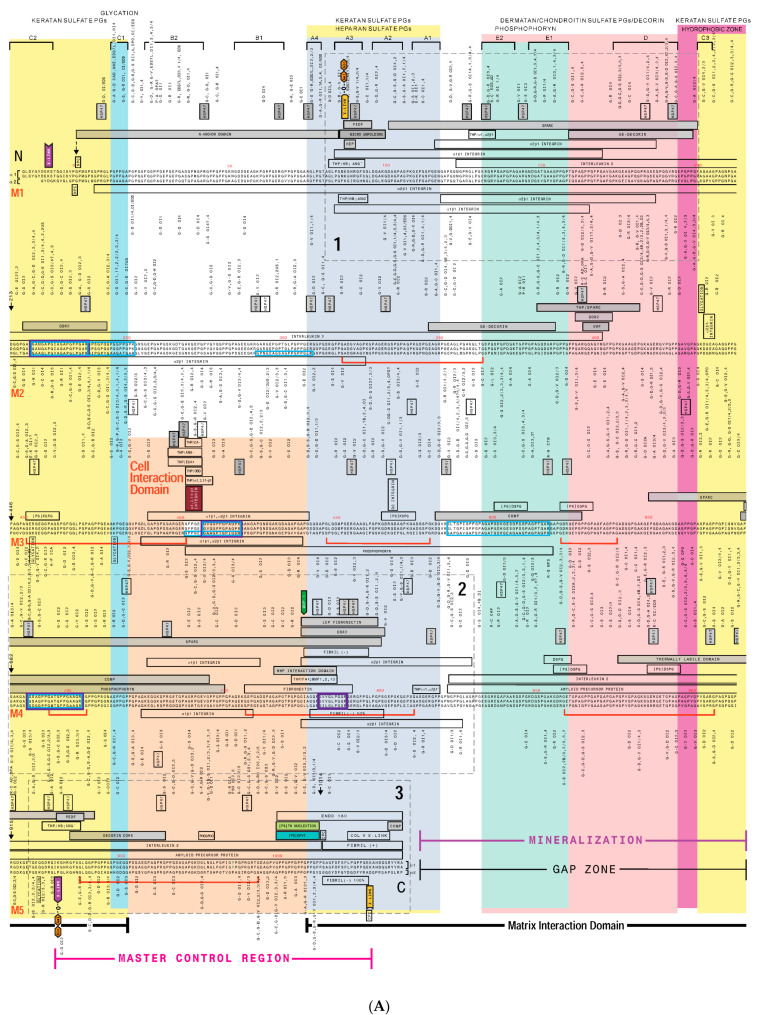

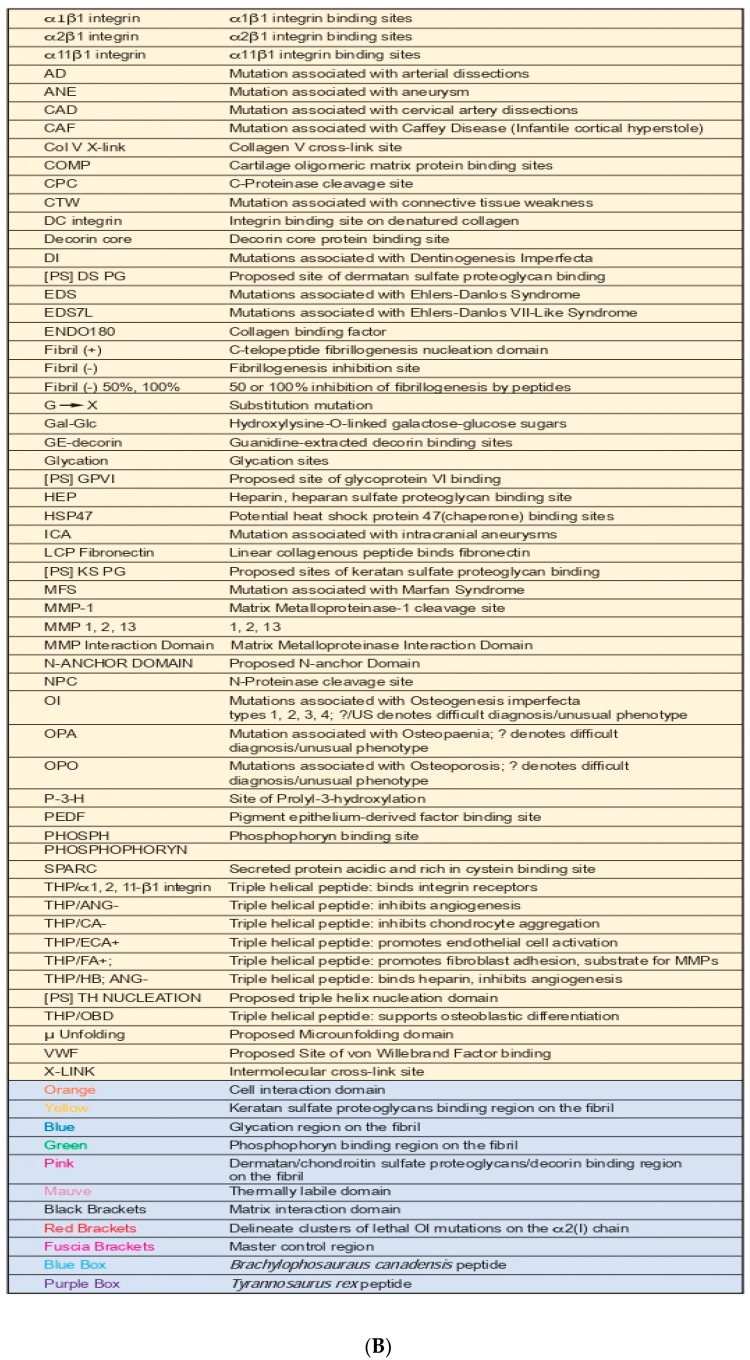
(**A**) Type I collagen interactome. Human collagen primary sequences were from GenBank, accession #s: a1(I), NP000079; a2(I), NP000080 and aligned as described [23] (Figure 1). Ligand binding sites are indicated by rectangular boxes adjacent to relevant collagen sequences. Gray boxes denote ligand binding to the monomer. Non-shaded boxes denote ligand binding to one α chain. Major ligand binding regions (MLBR) 1, 2, and 3 are designated. Disease-associated mutations are indicated next to affected residues. Broad cross-fibril ligand binding regions are delineated by color-shaded overlays. (**B**) Legend to type I collagen interactome (**A**) [22], listing abbreviations of mapped sites; literature citations are in [22]. Human mutation data date to 2013. This figure was modified from research originally published in the *J. Biol. Chem*. © the American Society for Biochemistry and Molecular Biology [22].

Over the past fifty years, a significant amount of information has been discovered about type I collagen, including its primary sequences, post-translational modifications, mechanisms of folding and polymerization, identification of its numerous binding partners, and mutations that result in a spectrum of diseases [1,3,5]. Twenty years ago, one of our labs began creating a type I collagen interactome in an effort to discover deep insights into the collagen structure-function relationship [22]. For example, we hoped to clarify the fundamental issues of whether type I collagen’s structural features, ligand binding sites, and mutations are distributed randomly or non-randomly on the molecule, and whether the protein has domains dedicated to specific functions. Furthermore, we wanted to know if we could correlate the positions of mutations in the collagen molecule and the disorders they cause, with their effect on protein expression or the disruption of a specific protein function. As the basis for the interactome, we used a model representing collagen monomer alignment within the fibril according to complementary electrostatic interactions, resulting in a molecular overlap of approximately a quarter stagger between the monomers, and zones of complete (overlap zone) and incomplete (gap zone) overlap [23] (Figure 1). Thus, on the interactome, the primary sequences of the α1(I) and α2(I) chains of five collagen molecules are arranged sequentially from the N-terminus to the C-terminus. The collagen molecule was then annotated with known functional sites such as those for intermolecular crosslinking, glycosylation, and matrix metalloproteinase-1 (MMP-1) cleavage. Next, the proposed binding sites for dozens of collagen-binding ligands such as cytokines, cell adhesion molecules, and proteoglycans were indicated. Finally, the positions of substitution mutations associated with human diseases including osteogenesis imperfecta (OI; brittle bone disease), Ehlers Danlos syndrome (EDS; although some EDS mutations map to type I collagen’s N-terminal region, most map to type III collagen as discussed later in this review), osteoporosis, and others were included (Figure 2). As an outcome, our interactome incorporated hundreds of ligand binding sites, functional domains, and human mutations [6,22].

A qualitative analysis of the collagen interactome revealed several observations. Particularly, ligand binding sites appear not to be randomly distributed as evidenced by at least three hot spots for ligand binding where many ligands exhibit overlapping, or in some cases, identical binding sites. On the other hand, some regions of the collagen fibril exhibit few or no ligand binding sites. Because of their prominence, ligand binding hot spots were named major ligand binding regions (MLBRs) [6]. Moreover, relatively more ligand binding sites located to the C-terminal half of the collagen molecule.

**Figure 3:**
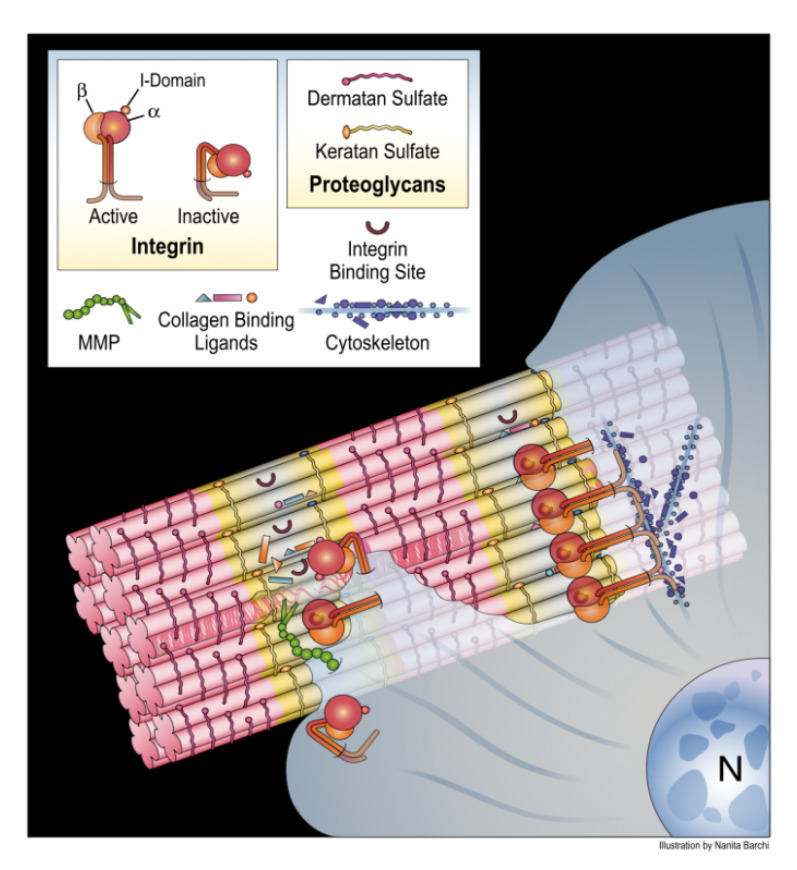
Domain model of collagen fibril function. Type I collagen interactome (Figure 2) and X-ray diffraction fibril model (Figure 4) suggests the conditional domain model of collagen fibril function. Depending on the physiological state of the tissue, the fibril predominantly supports structural duties including intermolecular crosslinking, proteoglycan binding, and biomineralization via the matrix interaction domain; alternatively, dynamic biological processes such as hemostasis, collagen remodeling, and cell adhesion are supported via the cell interaction domain, as schematically shown in Figure 5. This figure was originally published in the *J. Biol. Chem.* © the American Society for Biochemistry and Molecular Biology [22].

Multivalency in the binding of several ligands to collagen seemed to be a theme (e.g., for the secreted protein rich in aspartic acid and cysteine, SPARC [24]; discoidin domain receptor 2, DDR2 [25,26]; and integrin binding sites [27,28] as such ligands displayed multiple binding sites for collagen, each on different monomers of the fibril. Analysis of patterns of ligand binding sites and mutation distributions revealed the collagen fibril to comprise two distinct functional domains [22] (Figure 2 and Figure 3). Most of the dynamic aspects of collagen biology are proposed to occur in one region, called the “cell interaction domain”, which occupies much of the fibril’s overlap zone. This domain includes sequences mediating the binding of cell surface integrin receptors (a predominant mechanism of cell-collagen adhesion and interactions), various bioactive factors, collagen scission/remodeling by MMP-1 (vertebrate collagenase), angiogenesis, and hemostasis (blood clotting). The remainder of the fibril comprises the “matrix interaction domain”, proposed to assume a predominantly structural role, where intermolecular crosslinking between collagen monomers occurs, and PGs bind to regulate fibril solubility and inter-fibrillar spacing among other functions [19,29]. Furthermore, the matrix interaction domain likely harbors the putative sites where biomineralization initiates in tissues like bone. The mechanism of collagen biomineralization remains elusive; substantial data support three non-mutually exclusive hypotheses. The first maintains that the fibril contains several amino acid sequences and post-translational modifications that may potentially nucleate hydroxyapatite mineral [9,10,11,30,31,32,33,34,35,36]. Alternatively, several acidic non-collagenous bone phosphoproteins (e.g., SIBLING proteins such as osteopontin, bone sialoprotein, and phosphophoryn) are proposed to associate with collagen to provide the mineralization substrate [12,37]. Finally, it has been proposed that the spaces within and between the microfibrils may support biomineralization without the need for mineralization nucleation sequences or collagen-associated proteins [38,39,40,41].

### Human Mutation Patterns

The most abundant mutations of type I collagen are glycine substitutions in the *COL1A1* and *COL1A2* genes associated with OI, which is typically divided into four categories of disease severity: type I (mild), II (lethal), III (severe), and IV (moderately severe) [2]. This can be observed on the collagen interactome wherein the majority of mutations shown correspond to glycine (G) substitutions for another amino acid, as indicated above the triple helix for α1 chain mutations, or below for those on α2 (Figure 2). In addition, another category of OI mutations proposed to occur in type I collagen are “silent”, and assumed to have such severe consequences that affected individuals do not survive embryonic development, and are therefore not seen in the clinic [42]. The consequences of collagen substitution mutations are mainly proposed to arise via one of several means. The “gradient” model suggests that during its intracellular assembly, since the collagen triple helix folds from the C terminus to the N terminus, mutations located more C-terminal on the protein should affect collagen post-translational modifications and assembly more profoundly than those occurring in the N-terminal region, resulting in more severe phenotypes, which is typically observed [43]. Indeed, examination of the collagen interactome illustrates that OI mutations at the N terminus tend to be milder, and as one moves toward the C terminus the frequency of lethal mutations increases. In contrast, the “regional model” suggests that some mutations may directly disrupt specific functions of the mature protein resulting in disease pathologies [42,44]. Several possible examples of the latter were revealed by analysis of our collagen interactome. Thus, mutation silent zones appear on collagen monomer 3 (Figure 2) flanking the preferred binding site for αlβ1/α2β1 integrin receptors—GFOGER—and implying a critical functional role for this sequence [22]. Moreover, on the α2(I) chain, clusters of lethal OI mutations alternate with non-lethal ones, with the former corresponding to zones for the binding of PGs that play a crucial role in fibril structure and biology [2]. Further, some consecutive runs of glycine residues associated exclusively with lethal, non-lethal, or no mutations exist throughout the protein, in no discernable pattern. Many of the non-OI, or “atypical” mutations also do not exhibit a gradient of phenotype severity but rather, cluster to a few distinct zones on the fibril [22].

## 4. Translating the Collagen Interactome into the Three-Dimensional (3D) “Living” Fibril

The two-dimensional (2D) type I collagen interactome cannot distinguish between surface-exposed accessible sequences capable of interacting with cells and various bioactive factors, and inaccessible ones located internally within the fibril. Therefore, we will examine selected interactome data in the context of the X-ray diffraction model of the collagen microfibril created by one of our research groups. Before doing so, we will introduce the collagen microfibril structure in the context of the collagen fibril.

The collagen fibril is a crystalline construct, demonstrated by its ability to diffract X-rays particularly when extracted from a tissue in a hydrated, minimally-strained state [45,46,47]. The collagen microfibril model was determined from X-ray diffraction analysis of rat tendon type I collagen molecules in situ [18]. Fiber diffraction data collected from native and heavy atom-derivatized rat tail tendons were used to solve the structure of the collagen molecules via multiple isomorphous replacement [22]. A molecular model was constructed based on the primary sequences of the three α chains of type I collagen, and superhelical parameters were determined from structural analyses of triple helical peptide collagen models [18,48]. The electron density map representing the microfibril has a resolution of 0.516 nm along the fiber axis and 1.11 nm perpendicular to that. Translating the positions of functional sites from the collagen interactome to the three-dimensional microfibril model was accomplished by performing solvent-accessible surface calculations and rendering using SPOCK [49] with a 0.14 nm default probe size. The functional sites were color-highlighted within this representation. The nearly identical sequence identity between rat and human type I collagen justifies localizing functional sequences of human type I collagen on the rat-based collagen structure.

**Figure 4:**
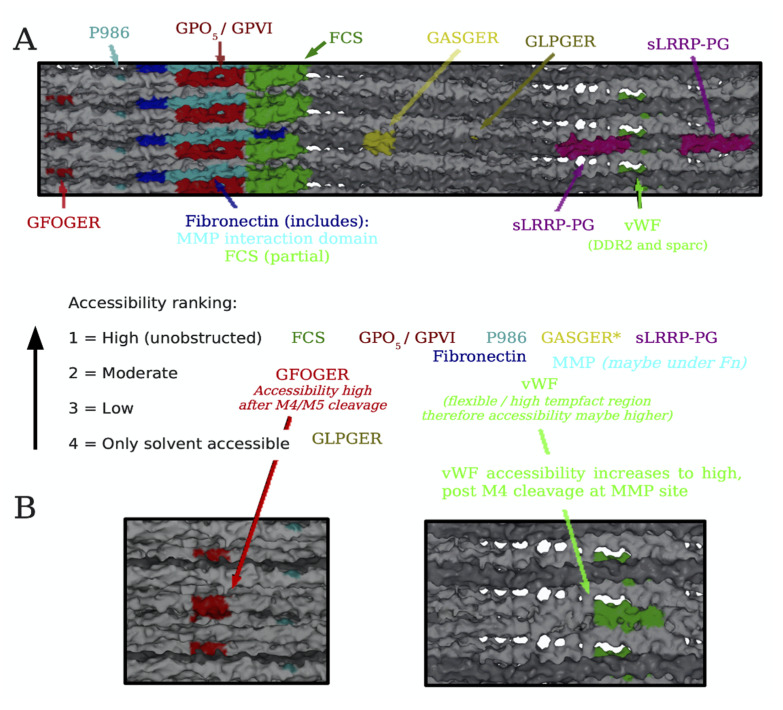
Ranking of ligand accessibilities to crucial binding sites and functional sequences in the native and proteolyzed type I collagen fibril. (**A**) Key functional domains of collagen were mapped onto a model of the fibril surface viewed from the fibril’s exterior. A molecular accessibility ranking of various ligands to their binding sites was determined for the static fibril, and following MMP-1 cleavage of M4 and “opening up” of the fibril. (**B**) View of the fibril’s GFOGER and Von Willebrand’s Factor (vWF)-binding sites following MMP-1 cleavage of M4. Reprinted with permission from [50].

Thus, to obtain a 3D, more physiologically relevant view of the collagen fibril we projected data from the interactome onto the collagen microfibril X-ray diffraction model [50] (Figure 4). Significantly, it was discovered that the domain model indicated by analysis of the interactome also held true for the native fibril. Next, we investigated surface-accessible and inaccessible regions of the fibril and their associated binding sites. Thus, surface accessibility of about a dozen functional sites was qualitatively ranked according to a four-tiered scale ranging from high, moderate, low, to least accessible to water molecules and small ions. For binding of ligands to the collagen fibrils, we identified some unobstructed sites, including glycoprotein VI (GPVI) [51], Endo180 (sequence required for endocytic recycling of collagen) [52,53], osteoclast-associated receptor (OSCAR; not shown on collagen interactome) [54], P986 (3-prolyl hydroxylation at amino acid position 986 required for collagen folding and post-translational modifications) [55], fibrillogenesis control sequences [56], residues mediating intermolecular crosslinking [3], and binding sites for cartilage oligomeric matrix protein (COMP) [57] and pigment epithelium-derived growth factor (PEDF) [58], which appear to co-locate at the interface of (GPO)_5_ at the C-terminal end of the triple helix and the start of the non-helical C-telopeptide [21,59]. Other unobstructed sequences of monomer 4 in the gap zone included the low affinity integrin binding sequence, GASGER [27], and the small leucine rich proteoglycan (SLRP) core protein binding sites [60] (Figure 4). Most of these sites are structural features involved in collagen folding, assembly, regulation of fibril solubility and interfibrillar spacing, and structural integrity. In contrast, (GPO)_5_ promotes hemostasis by ligating GPVI receptors on platelet membranes [51].

The relevance of the availability of GASGER, a relatively low affinity integrin binding site [27] is unclear. It would seem that platelets could bind GPVI and GASGER cooperatively based on their proximity [21]. Since the fibril surface within each D-period has an irregular topology with ridges and valleys akin to that of concrete rebar, we mapped the position of the most exposed functional sequences of collagen on the fibril surface; this revealed that the C-terminal portion of collagen is located at the apex of the fibril’s ridges, making it the exposed part of collagen, and thus freely accessible to cells and macromolecules [50]. Notably, the collagen sequences comprising the recessed portions or valleys of the fibril surface carry out structural duties, including sites for the binding of the decorin core protein [60], and in providing possible sites to nucleate and/or accommodate hydroxyapatite mineral growth in skeletal tissues. Thus, the exterior of the native collagen fibril projects a robust, structural face to the environment, yet is poised to promote hemostasis in response to tissue injury.

### Conditional Cell and Matrix Interaction Domains of the Type I Collagen Fibril

**Figure 5:**
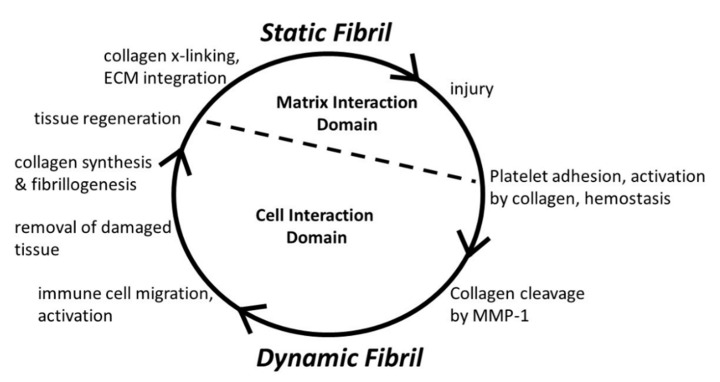
Schematic of conditional cell and matrix interaction domain model of type I collagen fibril function. See text and Figure 3 for details.

That these findings support a relatively stable, structural role for the collagen fibril is not surprising, but how can that view be reconciled with the abundant data in the literature showing collagen to be a dynamic substrate for cell-matrix adhesion, activation, and differentiation? The answer may come from collaborative studies by several of our labs, that analyzed the mechanism of collagen remodeling by MMP-1 [21,50,61,62] and determined that for collagen remodeling to occur, the fibril must be essentially “opened up” to render the MMP-1 cleavage site available for scission by the enzyme. For this to happen, the C-terminal telopeptide must first either be proteolytically removed by MMP-1 or another enzyme (e.g., telopeptidase), and/or pushed aside to expose the MMP-1 cleavage site on monomer 4 by either local mechanical action at the site on one or more molecules or via a larger tonal scale strain on the fibril/tissue [47]. Once monomer 4 is cleaved by MMP-1, other monomers with their host of binding sites, such as monomer 3 containing GFOGER, monomer 2 containing the Von Willebrand’s Factor (vWF), SPARC, and DDR2 binding sites become available to cells and bioactive factors. The domain model of the collagen fibril, when viewed through this lens, suggests that the cell and matrix interaction domains may be conditionally and mutually exclusively available according to the physiological state of the tissue in which the fibril resides, in a cyclic process (Figure 5). Thus, in a stable tissue, the static fibril will be dominated by structural functions of the matrix interaction domain. Tissue injury causes bleeding into the collagenous stroma, triggering the onset of the dynamic fibril where the functions of the cell interaction domain take over. Platelet adhesion to collagen via GPVI-(GPO)_5_ and possibly integrin receptor-GASGER ligations occur, leading to platelet aggregation, activation, and hemostasis. Release of MMP-1 from activated platelets and/or inflammatory cells causes telopeptide removal (or relocation) to expose sites for MMP-1 cleavage of collagen, and bioactive sites of the collagen fibril which are functionally crucial in the subsequent events. Moreover, as the fibril is further denatured and degraded, cryptic epitopes such as the pro-angiogenic sequence RGDKGE may be exposed [63], and bioactive collagen peptides [64,65] may be released into the tissue milieux which also promote the tissue regeneration cascade. Inflammatory cells are activated, enter the tissue, and migrate on the collagenous stroma in response to chemotactic factors and cytokines released by the activated platelets. The damaged tissue is removed by inflammatory cells, and tissue cells migrate and proliferate into the site of injury, while producing a new collagen-rich ECM which in turn promotes terminal tissue differentiation [14]. Integration and crosslinking of the newly synthesized collagen into a mature ECM culminates in re-emergence of the quiescent tissue state and the static fibril. For circumstances aside from tissue injury, such as bone remodeling or tissue growth, secretion of MMP-1 by cells such as osteoclasts or fibroblasts is also assumed to trigger collagen remodeling and initiation of the subsequent static to dynamic fibril cycle. Other possible mechanisms may also trigger the cycle such as cell-assisted mechanical relocation of the C-terminal telopeptide.

## 5. Type III Collagen Interactome

Like type I collagen, type III collagen also aggregates into a fibril. This collagen type plays crucial roles in embryogenesis [66], hemostasis [67], and wound healing [68,69]. Moreover, it is a vital element of blood vessels and other distensible organs. Collagen fibrils comprised solely of type III collagen are often found in tissues like the vasculature, but type III collagen also copolymerizes with type I and V collagens to form heterotypic fibrils in many tissues, including tendon, skin, and bone [70]. Mutations in the type III collagen human gene, COL3A1, may result in the vascular type of Ehlers-Danlos syndrome type IV characterized by widespread bruising due to rupture of blood vessels; this is the most serious type of EDS, since patients often die suddenly due to rupture of large arteries [71]. Researchers also showed that alterations of the type III: type I collagen ratio may be responsible for changes in the morphology of collagen fibrils seen, for example, in Achilles tendinopathies and in the fibrotic skin of lipodermatosclerosis [72,73].

**Figure 6:**
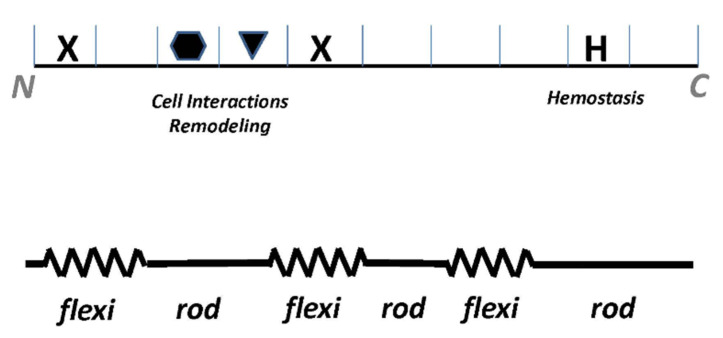
Analysis of type III collagen interactome suggests a “flexi-rod” model of fibril structure. Top: schematic of type III collagen fibril with sites for cell interactions and remodeling is flanked by intermolecular crosslinks (X) and a hemostasis domain (H). Bottom: Clusters of atypical collagen sequences of lower stability (springs) are interspersed with rigid zones (rods) hosting crucial biologic functions. Reprinted with permission from [74].

To learn more about the structure-function relationship for type III collagen and to relate this information to what we have gleaned for type I collagen, we constructed and analyzed an interactome of human-derived α1(III) homotrimer (not shown) [74]. Several main observations enabled us to validate our models for both type I and type III collagens. First, type III collagen has several of the same functional sequences and ligand binding hot spots as type I collagen, which occupy similar positions in the molecules; this can be seen by comparing the interactome of type I collagen (Figure 2) with that of type III collagen previously published (Figure 4) [74]. Thus, the domain model we proposed for type I collagen also holds for type III. However, as one would expect, type III collagen has more sites associated with hemostasis. Second, the nearly identical distribution of charged residues on type III and type I collagens suggests they polymerize in parallel to form heterotypic fibrils, resulting in the alignment of sites for cell adhesion, intermolecular crosslinking, and other functions in the composite molecule. Third, we discovered a possible explanation for why type III collagen has higher Gly, and lower Pro contents and about twice the number of “atypical amino acid triplets” (e.g., Gly-Ala-Ala or Gly-Gly-Y) compared with the other fibrillar collagens [3]. Since Gly-Pro-Pro triplets are the predominant contributors to triple-helix stability in fibrillar collagens, and atypical triplets are destabilizing [75,76], we used the collagen stability calculator [77] to plot the stability of the type III collagen molecule along its length according to its amino acid triple composition. We observed that in three locations on the type III collagen fibril, atypical amino acid triplets co-localized, predicting three major regions of decreased stability. We thus proposed a “flexi-rod” model for type III collagen in which confluences of atypical triplets create flexible domains, allowing focal expansion or deformation of several discrete fibril regions (Figure 6) [74]. The intervening rod-like regions adopt the rigid triple helical conformation and assume crucial functions like cell/ligand binding and proteolysis. Our model may explain how type III collagen’s unique complement of atypical amino acid triplets confers the molecular flexibility or pliability, a hallmark of the tissues in which this molecule predominates—such as embryonic or newborn skin, and distensible organs including blood vessels and the uterus.

## 6. Collagen Structure-Function Studies Inform Bioengineering Applications

### 6.1. Anti-Collagen Antibodies

In the context of fibril-ligand interactions, the C-terminal telopeptide region contains some of the most significant interaction sites [22,61,62]. As stated above, this is partly due to its occupying the “up” ridge of the pleated gap-overlap D-periodic structure, with the C-terminal telopeptide jutting out into the milieu (Figure 1 and Figure 4). It is also because the radial organization of the microfibril leaves the C-terminal telopeptides on the outside of the fibril [62]. Thus, this region appears available to interact with immunoglobulins and immunoglobulin-like receptors which may play prominent roles in matrix remodeling and antigen recognition [54,59]. The presence of these interaction sites “makes the C-terminal area of the mature collagen fibril an important region for receptor-mediated activation of cells, inflammation, cellular recruitment, and prevention of apoptosis” [59].

Recent studies raised antibodies against human collagen [78], that were later shown to bind the (GPO)_5_ sequence located at the junction of the triple helix and C-terminus, revealing novel immunologically relevant properties at the gap-overlap interface [59]. The binding brought the antibody into the range of the tyrosine residues found exclusively within the telopeptides. This is significant due to the importance of tyrosine to the function of OSCAR, an immunoglobulin-like activating receptor of the leukocyte complex [54,79]. This receptor is expressed at high levels in osteoclasts. When bound to collagen, OSCAR triggers several signaling pathways involved in the modulation of matrix remodeling and immune cell behaviors. Similar to the anti-(GPO)_5_ antibody, modeling of OSCAR binding to collagen demonstrated preference for the same binding site.

### 6.2. Collagen Fibrillogenesis as a Potential Anti-Fibrotic Target

Knowing specific roles of collagen molecules and fibrils allows their targeting to achieve therapeutic effects. For example, one of our research groups targeted collagen fibrillogenesis to block the growth of fibril-rich scars formed in response to injury [80,81]. While fibrillogenesis is a crucial element of the mechanism that maintains physiological homeostasis of connective tissues, excessive collagen biosynthesis, and accelerated fibril formation define fibrotic diseases. Regardless of the etiology and tissue-specific pathways of these diseases, it is collagen-rich fibrillar deposits that disrupt vital functions of fibrotic tissues and organs. For instance, in pulmonary fibrosis, dense collagen-rich tissue obstructs the exchange of gases, thereby limiting blood oxygenation [82]. Moreover, excessive formation of collagen-rich scars in the skin, peripheral nerves, vocal cords, cornea, and other tissues severely limits their vital functions [83,84,85,86].

Currently, there are no therapeutics available that reduce fibrosis effectively and safely. The main targets that may limit the fibrotic response to tissue injury include pro-fibrotic inflammation and activation of pro-fibrotic cells. These targets are nonspecific intracellular processes that control not only the accelerated production of elements of the scar tissue, most notably collagen, but also other processes not associated with fibrosis. In an attempt to target fibrosis specifically and safely, one of our research groups identified collagen fibrillogenesis as an attractive anti-fibrotic target [80]. The rationale for selecting fibrillogenesis was that, regardless of the type of injury or its anatomical location, collagen fibrils are the main element of the fibrotic tissue formed due to excessive scarring. This notion is illustrated by the fact that collagenous fibrils comprise over ninety-five percent of the dry mass of pulmonary scars [87]. To attack fibrillogenesis therapeutically, our group selected collagen telopeptides as the ultimate target. As demonstrated by various studies, telopeptides drive the collagen-collagen binding interaction that plays a crucial role in the nucleation and growth of the fibril [56,88]. Thus, blocking telopeptide-mediated interactions inhibits collagen fibril formation in a concentration-dependent manner [80,89]. Subsequently, utilizing a de novo fibril formation experimental system, we showed that a monoclonal antibody targeting an epitope within the C-terminal telopeptide of the α2(I) chain—its C-terminal telopeptide (α2Ct)—inhibits fibril formation in vitro and in an organotypic model of skin fibrosis [80,89]. These observations strongly suggested that blocking type I collagen fibrillogenesis may reduce the formation of fibrotic tissues in vivo. More recently, we confirmed the anti-fibrotic effects of the anti-α2Ct monoclonal antibody in vivo [80,81]. Moreover, we demonstrated that the anti-α2Ct antibody binds both free, intact type I procollagen and type I collagen molecules [80]. Consequently, this antibody inhibits the nucleation step in fibril formation by blocking the assembly of collagen monomers. Its interaction with mature fibrils, however, is less understood. One study that utilized type I collagen fibrils formed de novo demonstrated, however, that the antibody interacted with the gap region of the type I collagen fibril [89]. Since these fibrils were formed in vitro and contained type I collagen only, there were no other macromolecules present that could block the access to the gap region in the fibrils, making the in vivo relevance of these data unclear.

In the context of its anti-fibrotic application, determining potential mechanisms of the anti-α2Ct antibody’s binding interactions with the collagen fibrils is crucial because: (i) binding of the antibody to growing, immature fibrils would inhibit their further growth, and (ii) binding of the antibody to the mature fibrils could reduce its bioavailability. Thus, we carried out molecular binding simulations between the α2Ct antibody and C-terminus of type I collagen as detailed below.

### 6.3. Modeling of Anti-α2Ct Antibody-Collagen Fibril Interactions

#### 6.3.1. Collagen C-Terminus

**Figure 7:**
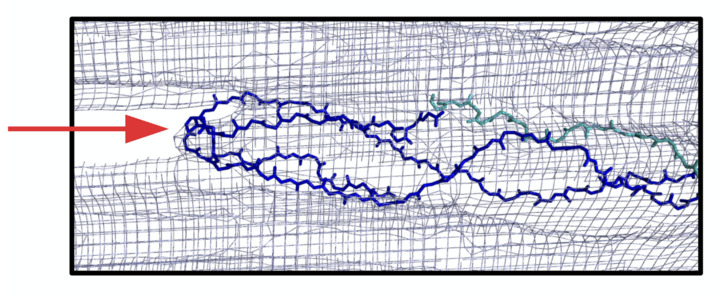
X-ray diffraction model of the C-terminal telopeptide region of the rat type I collagen microfibril. The truncated α2(I) chain is shown in cyan (see text). The electron density defining the C-terminal region is seen to terminate at the ends of the C-telopeptides, where the α1(I) chains, in dark blue, fold back on themselves. The anti-fibrotic antibody binding sequence on human collagen—the α2Ct epitope—can be accommodated within the limits of the C-terminus of the rat protein, however the homologous sequence on the rat α2(I) chain is not found in the structural electron density model (gray wireframe, C-terminal electron density indicated; red arrow). To enable molecular modeling of scFv antibody-collagen interactions with rat collagen, the α2(I) chain of the rat protein was extended C-terminally to include the α2Ct epitope of the rat/human sequence in the molecular model shown in Figure 8 (see text and Table 1).

To illustrate the possible interaction between the anti-α2Ct antibody and the C-terminus of human type I collagen, we employed the rat tendon X-ray diffraction model of the type I collagen fibril published by one of our groups [18]; however the predicted epitope, or sequence for the binding of the antibody to the α2Ct of the rat protein is not a perfect match with that of the human protein against which the antibody was raised (Table 1). The sequences are reasonably homologous between rat, human, and other vertebrates commonly used in research (Table 1); however, the α2Ct epitope of the human protein GGGYDFGYDGDFYRA, appears to be truncated in the X-ray diffraction model of the rat protein to: xGGYDF (where x is either G or S). The epitope is however clearly located at the exterior of the collagen fibril in the highly exposed C-terminus as expected. Although the molecular sequence database indicates that the α2 rat sequence is longer than that seen in the molecular model of the rat collagen microfibril, the electron density near the C-terminus is only well-defined around the α1 chains (Figure 7). This indicates that the α2 chain in the rat is not as long as the sequence data suggests, or that its full sequence is present in the fibril but that its C-terminus is significantly disordered and not discernible by X-ray diffraction. Thus, for the purposes of this review we compensated for these discrepancies by extending the rat α2 chain by nine amino acids GFEGGFYRA from the rat sequence (Table 1) in our modeling simulations. Even in an extended and unfolded confirmation, we observed that the α2Ct epitope is the most exposed of all of the α chains (Figure 7), while still in position to precisely define the interface between the gap and overlap zones of the fibril [18]. Therefore, on balance of these considerations, the α2Ct epitope appears to be fully available on the collagen fibril for interactions with macromolecules such as a single chain variable fragment (scFv) variant of the anti-α2Ct antibody. Even if in reality there is some variation, disorder, or if “tucked” alongside one of the α1 peptides, the telopeptide site on the outside of the fibril is relativity uncluttered and should still present a highly viable target for the antibody.

#### 6.3.2. Antibody Variant Used for Modeling

**Figure 8:**
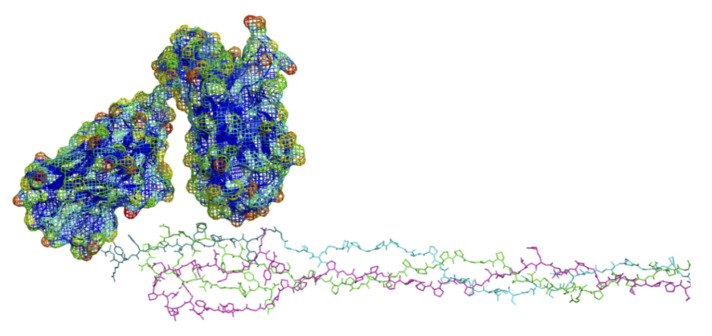
Preliminary molecular simulation of docking between the scFv antibody and the α2Ct epitope of rat collagen. Lower figure: C-terminus of the rat type I collagen model; α1(I) chains, purple and green; α2(I) chain, cyan, α2Ct epitope, dark cyan. Upper figure: scFv antibody with highly water accessible “exposed” regions (mesh surface) color-scaled towards the red end of the spectrum; green is intermediate and blue is not exposed. Antibody subunits show potentially complementary binding interactions at α2Ct epitope (see text).

Since the crystal structure of the anti-α2Ct antibody is unavailable, modeling its binding interaction depends on homology-based structural models. Here, we applied a molecular model of a mini-version of the anti-α2Ct antibody, the scFv. A linker connects its fragments, corresponding to the original complementary deter-mining regions [89]. Thus, the scFv includes the same domains for epitope binding as the native antibody, as well as exhibiting comparably selective, high affinity binding to the α2Ct of collagen [89]. Consequently, the scFv offers an excellent model to represent the anti-α2Ct antibody-α2Ct binding interaction in the context of the collagen fibril. The scFv SwissProt-generated homology model was obtained without modification. The highly water-accessible “surface exposed” residues within the variable loops of the antibody were bought into contact with the molecular dynamics-generated α2Ct epitope of the collagen C-telopeptide (Figure 8). There appears to be ample potential for complementary surface interactions between the molecules, suggesting the α2Ct epitope may serve as a candidate binding site for the scFv antibody. Further computational studies are required to establish the potential molecular bases for such a binding interaction, and, if such exists, to confirm the model experimentally. Based on these preliminary data, we speculate on how the scFv antibody could impede collagen deposition in fibrotic scars in vivo, by blocking the attachment of additional collagen molecules to the surfaces of growing fibrils, e.g., via disruption of the function of the fibrillogenesis control sequences [56] (Figure 2 and Figure 4). In addition to impeding the growth of the fibrils, the antibody-telopeptide interaction may also prevent the formation of new fibrils by blocking telopeptide mediated collagen-collagen aggregation during the nucleation phase of fibrillogenesis [56]. Considering that the full-length antibody is about 150 Å in diameter and much greater in size than the scFv, its interaction with the α2Ct may render neighboring binding sites on the fibril inaccessible; i.e., it may inhibit other collagen functions aside from fibrillogenesis. Consequently, binding of the anti-α2Ct antibody may not only exert its anti-fibrotic effect via direct blocking of collagen fibrillogenesis but also by disrupting other mechanisms underlying complex scar formation.

### 6.4. A Broad View on Blocking Matrix Assembly as an Anti-Fibrotic Therapy

The model we propose for the anti-α2Ct antibody-collagen fibril interaction would provide a blue print for other interactions with anti-fibrotic potential. We realize that although type I collagen is the main component of fibrotic deposits, other collagenous and non-collagenous macromolecules also contribute to the fibrotic mass. For instance, the production of fibronectin is also upregulated in fibrotic processes, and its assembly may precede that of collagen fibrils. Consequently, a peptide inhibitor of fibronectin fibrillogenesis has been shown to exhibit anti-fibrotic properties [90]. Other compounds were also suggested as inhibitors of collagen fibrillogenesis, and potentially, anti-fibrotic therapeutics. In one example, researchers employed α-lipoic acid to block a site-specific interaction that drives collagen fibril assembly [91]. In similar experiments, trigonelline hydrochloride was shown to be an effective inhibitor of collagen fibrillogenesis [92]. Recently, two small molecules, nintedanib, and pirfenidone, were approved for the treatment of idiopathic pulmonary fibrosis (IPF). Although their mechanism of action is not clearly established, nintedanib is a receptor kinase inhibitor of platelet-derived growth factor receptor and vascular endothelial growth factor receptor, all of which play roles in the pathology of IPF. Pirfenidone is an anti-fibrotic, anti-inflammatory, and antioxidant compound with beneficial effects in organ fibrosis, but its direct targets are not known. In addition to the cellular effect of these compounds, however, it was demonstrated that they directly block collagen fibril formation [93]. Furthermore, since type I collagen molecules copolymerize with type III and type V collagens to form heterotypic fibrils in vivo, we cannot exclude the possibility that the anti-αCt antibody disrupts the co-assembly process [94,95]. These examples further justify targeting the assembly of collagen-rich matrices as potential anti-fibrotic therapies.

## 7. Collagen-Induced Angiogenesis

In adult mammals, angiogenesis—or new capillary growth from an existing vasculature—is the single mechanism by which new blood vessels develop [96]. Angiogenesis is involved in homeostasis and diseases, including for example, solid tumor growth and metastasis, rheumatoid arthritis, hemangioma formation, and diabetic retinopathy [96]. It is a complex process by which endothelial cells degrade the surrounding ECM, migrate, proliferate, and differentiate to form new vessels [97]. Angiogenesis depends upon the interaction of endothelial cells with the ECM via cell surface adhesion molecules including the integrins, and the activities of growth factors and cytokines [98]. Because type I collagen is a ubiquitous component of many tissues that undergo angiogenesis during embryogenesis, it may play a role in promoting normal as well as pathological angiogenesis. For example, in vivo, angiogenesis is disrupted in the chick embryo by inhibiting collagen triple helix formation or fibrillogenesis, using several chemical inhibitors of those processes. In fact, type I collagen has also been demonstrated to be among the few optimal scaffolds for the induction of angiogenesis in vitro. Thus, type I collagen synthesis by endothelial cells [99,100] precedes angiogenesis, and is limited to the vicinity of capillary tube formation in cultures [101,102]. Furthermore, endothelial cells grown between or within collagen gels rapidly form capillary tube networks [103,104].

### Integrin Receptor Ligation and Clustering in Collagen-Induced Angiogenesis

**Figure 9:**
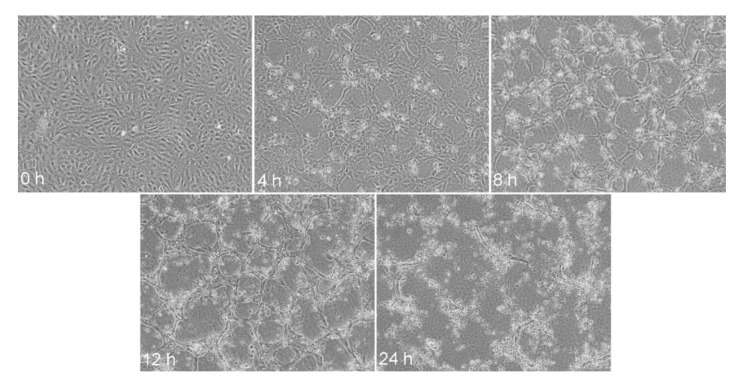
Apical collagen gel induces angiogenesis of human endothelial cells. Confluent monolayers of cells were overlain with a type I collagen gel at 0 h. At 2–4 h, cell streaming and reorganization occurred. At 6–8 h, cultures were at least about 50% reorganized and at 12–24 h, capillary tube formation was complete. Bar = 50 μm. Reprinted with permission from [106].

**Figure 10:**
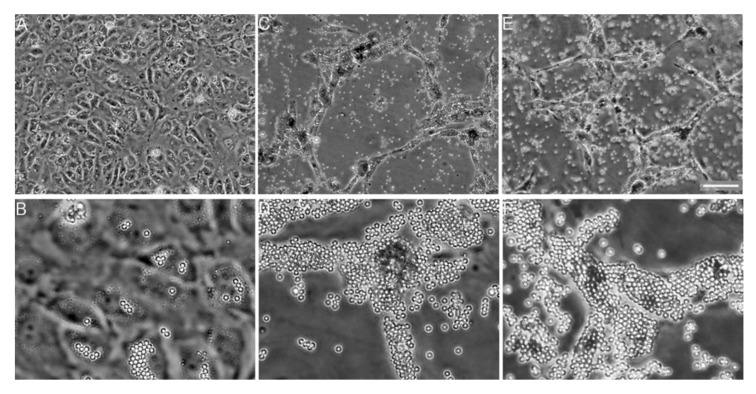
Anti-integrin antibody coated beads induce angiogenesis in the absence of collagen. Endothelial cells were exposed to 0.5 × 10^7^ beads/cm^2^ polystyrene beads (3 μm diameter) coated with bovine serum albumin as a negative control (**A**,**B**), anti-α2β1 integrin antibodies (**C**,**D**), and collagen (**E**,**F**). At 24 h, monolayers were rinsed and photographed. BSA beads showed no activity (**A**) and interacted with cells poorly (**B**). Anti-α2β1 integrin antibody-coated beads induced tube formation similar to that of an apical collagen gel ((**C**), see Figure 9) and interacted extensively with cells (**D**). Collagen-coated beads induced angiogenesis (**E**) and also interacted extensively with cells (**F**). Top row: Bar = 100 μm. Bottom row: 20× objective. Bead diameters = 3 μm. Reprinted with permission from [106].

Our research showed that in vitro, apical type I collagen gels rapidly induce angiogenesis in human endothelial cell monolayers (Figure 9) [105]. Further, angiogenesis required the engagement between the α2β1 integrins of the endothelial cells and the central integrin binding site, GFOGER, on the collagen molecule [105]. Further studies demonstrated that in quiescent endothelial cell confluent monolayers, in the absence of exogenous collagen, the α2β1 integrins concentrated along cell-cell borders. After adding apical collagen gels to the cultures, the integrins redistributed to apical cell surfaces, aligning with collagen fibers, which also relocated during angiogenesis [106]. Based on these observations, as well as on the distribution of integrin binding sites in the type I collagen fibril, we proposed that the fibril may function as an ideal substrate for integrin receptor clustering and activation necessary for angiogenesis [7,106]. Therefore, we next tested whether the polyvalency of collagen fibrils could be mimicked using antibody-coated polystyrene beads to cluster endothelial cell surface integrins, and thereby induce angiogenesis in the absence of collagen. It was discovered that clustering of α2β1 integrins, as well as αvβ3 integrins and PECAM-1, but not of α1 integrins, induced rapid angiogenesis even in the absence of collagen, whereas those same antibodies, when added alone to cultures, had no effect (Figure 10) [106]. Thus, the angiogenic property of type I collagen may reside in its ability to bind and cluster cell surface α2β1 integrins as well as select other endothelial cell surface receptors.

## 8. Bioengineering Applications: Angiogenic Polymers

We previously reviewed strategies to produce angiogenic polymers based on our understanding at that time of the collagen structure-function relationship and mechanisms of angiogenesis [7]. Here we will update and expand upon some of those strategies accounting for the newer information on these topics. As pointed out previously, type I collagen from animal sources is an effective angiogenic polymer. Moreover, collagen is easy to isolate in the lab but is also commercially available at reasonable cost. The collagen preparations we used to promote angiogenesis consisted of acid soluble, non-pepsinized, type I collagen from rat tail tendon that were applied to endothelial cells as gels. The angiogenic properties of collagen required its native, triple helical, and fibrillar conformations [105]. According to the domain model of collagen fibril function, for a collagen fibril to support angiogenesis, it must have an accessible MMP-1 cleavage site, which must be cleaved by MMP-1 released by endothelial cells to expose the underlying central integrin binding site, GFOGER. Indeed it has been reported that growth factor-dependent angiogenesis in vivo requires MMP-1-mediated cleavage of collagen [107]. Collagen preparations of the type we used are expected to have most of their telopeptides, with a relatively low density of intermolecular crosslinking at those regions which should facilitate their removal by MMP-1. Such non-pepsinized, telopeptide-containing collagen is preferred as an angiogenic polymer since the presence of telopeptides allows for rapid fibrillogenesis and robust gel formation in comparison with atelopeptidic collagen produced through pepsinization, because the latter may not undergo fibrillogenesis or gelation adequately [3].

### 8.1. Recombinant Super-Angiogenic Collagens

To engineer a super-angiogenic polymer, one may consider creating recombinant collagens with an enhanced capacity to ligate and cluster endothelial cell integrin receptors. The most obvious way to do so would be to add integrin binding sequences to surface-exposed regions of the fibril; based on our structural mapping, those could be placed within monomer 5 and the gap zone portion of monomer 4. Given the diameter of the integrin heterodimer of about 10–20 nm, only two or three such receptors could simultaneously ligate the fibril and be accommodated within the 67 nm D-period. Since there is only one low affinity integrin binding site on the fibril surface—GASGER, that sequence should be replaced by the high affinity integrin binding sequence GFOGER. Possibly, up to two other GFOGER sequences might be inserted elsewhere in the exposed fibril regions, taking care not to disrupt other functional sites or residues crucial for fibril assembly and integrity (see Figure 2 and Figure 4). Other type I collagen sequences that should not be removed or altered in the recombinant protein include those proposed necessary for the angiogenic properties of collagen: the MMP-1 interaction domain; the MMP-1 cleavage sequence; the central integrin binding site GFOGER; and the N- and C-terminal heparin binding sites. Functional sites that may be removed or modified to render them inactive include those for the binding of SPARC, PEDF, and decorin, all of which may exhibit anti-angiogenic activities [108,109,110,111]. Including C- and N-telopeptides in the polymer would be advantageous for rapid fibrillogenesis and robust gel formation properties of the material, but removing the capacity for intermolecular crosslinking within the C-terminal telopeptide is suggested, to render the polymer more easily proteolyzed by MMP-1, a proposed step in angiogenesis induction by collagen.

### 8.2. Collagen Mimetics as Angiogenesis Substrates

The discovery and application of collagen mimetics is an emerging field in biotechnology, and is reviewed extensively by Xu and Kirchner in this issue. The “bare bones” of the collagen fibril vis-a-vis its angiogenesis promoting activity appears to be its ability to ligate, and by virtue of its multivalency, cluster α2β1 integrin receptors. Theoretically, several classes of novel materials may mimic that activity and substitute for collagen. One class comprises two versions of synthetic “sticky-ended” collagen-like peptides that self-assemble into triple helices and higher ordered structures resembling collagen fibrils, and in one case also forms stable hydrogels [112,113]. The peptides can carry the high affinity integrin binding site GFOGER crucial for angiogenesis promotion, as well as the sequence of type I collagen rendering the polymers sensitive to MMP-1 degradation; the latter would ensure its shorter in vivo half-life if desired. Such peptides could also be modified to promote hemostasis instead, by carrying GPO_5_, and in some cases, also GFOGER if both hemostatic and pro-angiogenic activities are desired. A second class of collagen mimics is suggested by our in vitro angiogenesis studies [106]. Thus, biodegradable beads (e.g., albumin, polycaprolactone, or poly(D,L-lactide-coglycolide) among others) of about 1–5 μm diameters and onto which anti-α2β1 integrin antibodies are adsorbed or covalently linked may also mimic the angiogenic activities of collagen. Instead, it may be of interest to affix anti-α2β1 integrin antibodies at high densities on biodegradable fibrous polymer filaments to achieve a more collagen-like substrate for angiogenesis. The angiogenic collagen mimics described here may in several respects be preferable to using native or recombinant collagen as angiogenic substrates to avoid the drawbacks of using large, complex proteins from natural sources, including the presence of undesirable binding activities, batch-to-batch variations, and potential immunogenicity, among other considerations.

## 9. Summary

Here we attempted to reconcile the biology of the type I collagen fibril with its complex three-dimensional structure. Data suggest that in quiescent tissues, the fibril assumes structural duties, but tissue trauma may lead to collagen proteolysis, exposing a host of cell- and ligand-binding sites crucial for tissue regeneration. Moreover, our research helped identify a possible mechanism of action of an anti-fibrotic collagen-binding antibody, and develop non-collagenous agents that mimic collagen’s ability to cluster endothelial integrins and promote angiogenesis. Further structure-function analysis of collagen fibrils in various physiologic contexts will help elucidate poorly-understood aspects of collagen biology like fibril assembly and biomineralization, and facilitate the discovery of novel therapies to combat human pathologies where collagen plays prominent roles.

## Figures and Tables

**Table 1:** Ct domain from six vertebrates.

*Homo sapiens* *	GGGYDFGYDGDFYRA
*Oryctolagus cuniculus* *	GGGYDFGYDGDFYRA
*Macaca nemestrina* *	GGGYDFGYDGDFYRA
*Rattus norvegicus*	GGGYDFGFEGGFYRA
*Mus musculus*	GGGYDFGFEGDFYRA
*Gallus gallus*	GGGYEVGFDAEYYRA

* Putative binding epitopes for the anti-α2Ct antibody, underlined. Sequences are from the National Center for Biotechnology Information (USA).

## Data Availability

The collagen data is housed in the RCSB database under code 3HR2.
